# Doppler and birth weight Z score: predictors for adverse neonatal outcome in severe fetal compromise

**DOI:** 10.1186/1476-7120-5-15

**Published:** 2007-03-20

**Authors:** Fernanda C da Silva, Renato A Moreira de Sá, Paulo RN de Carvalho, Laudelino M Lopes

**Affiliations:** 1 Universidade Federal Fluminense, Niterói – Brazil; 2 CPDT – Laranjeiras Clínica Perinatal, Rio de Janeiro – Brazil

## Abstract

**Background:**

An adequate placental perfusion is crucial for the normal growth and well being of the fetus and newborn. The blood flow through the placenta can be compromised in a variety of clinical situations, always causing important damage to the gestation. Our objective is to identify significant predictors for adverse neonatal outcome in severe fetal compromise.

**Methods:**

Consecutive premature fetuses at between 25 and 32 weeks with severe placental insufficiency were examined prospectively. Inclusion criteria were: (i) singletons (ii) normal anatomy; (iii) abnormal umbilical artery Doppler pulsatility index (PI); (iv) abnormal cerebroplacental ratio; (v) middle cerebral artery (MCA) PI < - 2SD ("brain sparing"); (vi) last Doppler examination performed within 24 hours prior to delivery. All 46 patients that met criteria and started the study were followed to the end. We considered as independent potential predicting variables: absent or reversed end diastolic flow in umbilical artery, abnormal ductus venosus S/A ratio, absent or reversed flow during atrial contraction in the ductus venosus and birth weight Z score. Outcome parameters were: neonatal mortality and severe neonatal morbidity.

**Results:**

Backward stepwise logistic regression analysis was used to determine the optimal model for the prediction of neonatal mortality and severe neonatal morbidity. In this analysis birth weight Z score index showed the strongest association OR = 1,87 [1,17-2,99] with all neonatal outcome, all other independent variables were excluded for the optimal model. There was no mortality for the group with normal birth weight Z score.

**Conclusion:**

Our study suggests that birth weight Z score is the strongest predictor of adverse neonatal outcome in severe placental insufficiencies. Such use of Z scores, allowing to get rid of gestational age or sex covariates could be extended to estimated fetal weight and might help in making important decisions in the management of compromised pregnancies.

## Background

An adequate placental perfusion is crucial for the normal growth and well being of the fetus and newborn. The blood flow through the placenta can be compromised in a variety of clinical situations, always causing important damage to the gestation.

Placental insufficiency promotes compensatory hemodynamic fetal changes including blood flow redistribution towards essential fetal organs, at the expense of others [1]. The fetal compensatory response results in increased blood flow to the brain, also called the "brain sparing effect" [2]. On the other hand, there is reduction in fetal growth, of liver size, and a reduction or absence of fat deposit. As the placental disease progresses, however, the fetus no longer can keep his vital organs functioning, ultimately leading to severe compromise with acid-base disturbance and death.

Doppler analysis, mainly the umbilical artery indices, signals the malfunctioning of the placenta. Doppler can monitor the fetal hemodynamic changes, as the more the fetus is compromised, the more the arterial and venous flow is deteriorated. Nevertheless, Doppler examination is not absolute and additional information is necessary to adequately assess fetal status [3].

The challenge in monitoring pregnancies complicated by placental insufficiency remains today, as no method of diagnosis or follow-up is complete. The dilemma involves essentially premature babies since the effects of prematurity need to be highly considered. Research is still needed to help finding the best time of delivery, when the effects of fetal hipoxia become worse than those of the low gestational age and weight.

Gestational age at delivery showed strong association with all postpartum complications [3]. Using Z score indices, gestational age effect and gender effect can be removed. Our hypothesis is that Doppler and birth weight Z score index must be important to predict neonatal outcome. The aim of this investigation was to examine the relationship between, Doppler, birth weight Z score and adverse neonatal outcome in severe fetal compromise.

## Methods

### Patients

Between November 2003 and December 2006, all patients referred, between 25 to 32 weeks, with severe fetal compromise were examined prospectively. Inclusion criteria were: (i) singletons (ii) normal fetal anatomy; (iii) umbilical artery Doppler pulsatility index (PI) more than 2 standard deviations (SD) above the gestational mean values [4]; (iv) abnormal cerebroplacental ratio (middle cerebral artery pulsatility index divided by umbilical artery pulsatility index) [2]; (v) middle cerebral artery (MCA) PI more than 2SD below the gestational age mean ("brain sparing") [5]; (vi) last Doppler examination performed within 24 hours prior to delivery. Gestational age was determined by last menstrual period and/or sonographic examination prior to 20 weeks of gestation [6]. All 46 consecutive patients that met inclusion criteria agreed participating in the study, by signing a written informed consent, and none were lost in follow up.

This protocol was approved by Hospital Ethics Comitee (protocol number 08/2005).

### Ultrasound examination

**Figure 1:**
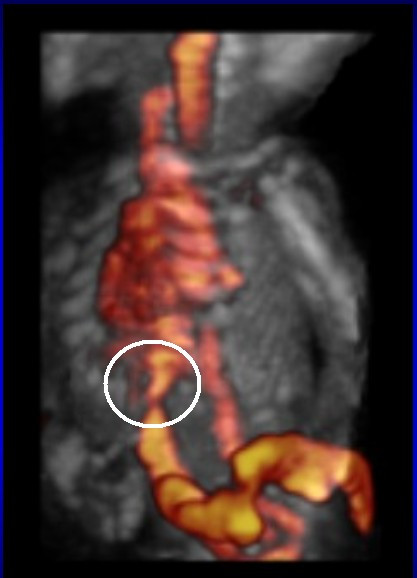
Highlighting of ductus venosus.

**Figure 2:**
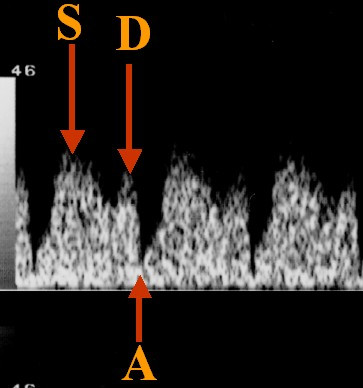
Normal ductus venosus wave.

For all ultrasound examinations 4 or 5 MHz sector ultrasound transducer (Voluson 730, GE Medical Systems, USA) were used, with spatial peak temporal average intensities below 10 mW/cm2 and the high pass filter at 100 Hz. During each examination Doppler measurements were obtained from the umbilical artery (UA), MCA and ductus venosus (DV) by previously described methods [4,7-9] (Figure 1, 2). Color Doppler imaging was used to optimize placement of the pulsed wave Doppler gate by adjusting the velocity scale to identify the area and direction of maximum blood flow. The insonation angle was kept as close to 0 as possible and the sample volume was adjusted to cover the entire vessel. Measurements were taken from the frozen image after at least five consecutive uniform flow velocity waveforms with a high signal to noise ratio were obtained during periods of fetal rest and apnea [3].

Z scores according to the reference charts for estimated fetal weight [10] were calculated in all cases using the following formulae:

*Z score *= (*X*_*GA *_- *M*_*GA*_)/*SD*_*GA*_

Where X_GA _is the measured birth weight at a known gestational age (GA), M_GA _is the mean value according to the reference used at this GA and SD_GA _is the standard deviation of the mean value at this GA according to the reference.

**Figure 3:**
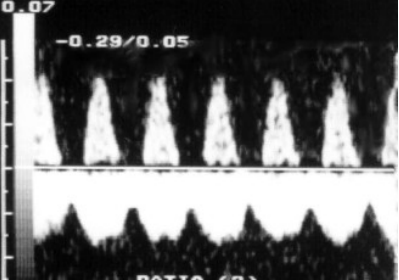
Absent end diastolic flow in umbilical artery.

**Figure 4:**
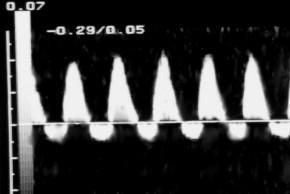
Reversal end diastolic flow in umbilical artery.

**Figure 5:**
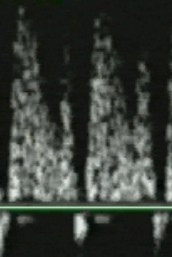
Reversal flow during atrial systole in ductus venosus.

We considered as independent variables absent or reversal end diastolic flow in umbilical artery (Figure 3, 4), abnormal ductus venosus S/A ratio [11], absent or reversal flow during atrial systole in ductus venosus (Figure 5) and abnormal birth weight Z score index.

### Neonatal Outcome

Outcome analysis was limited to the neonatal period. Outcome parameters were: neonatal mortality and severe neonatal morbidity. The clinical course at the neonatal period was reviewed for all surviving infants. The criteria for classification as severe neonatal outcome are the presence of one of following diagnoses: periventricular-intraventricular hemorrhage (PIH) grade 3 or 4, retinopathy of prematurity (ROP) stage 3 or 4, cystic periventricular leukomalatia (CPL) or broncopulmonary dysplasia (BPD) [12].

### Statistics

Stepwise logistic regression analysis was performed to determine the optimal model for the prediction of neonatal outcome. The Fisher's exact test was used to compare the frequency of outcomes between groups. The area under receiver-operator characteristic (ROC) curve was calculated for the significant independent variable, p < 0.05 was considered statistically significant.

All statistical analyses were performed using SPSS for Windows, version 10.0 (SPSS, Inc., Chicago, Illinois).

## Results

The study period was from November 2003 to December 2006. The mean gestational age at Doppler imaging was 28 weeks gestation, with the mean interval from Doppler imaging to delivery of 3 days.

All forty-six patients that initiated the study were included in the follow up. In 34 fetuses (74%) there was absent or reverse end-diastolic flow in umbilical artery. Nineteen fetuses (41%) had an abnormal ductus venosus S/A ratio. In 04 fetuses (9%) there was absent or reversal flow during atrial systole in ductus venosus. Twenty-two fetuses (48%) had birth weight Z score index below lower limit (-1.645).

We used backward stepwise logistic regression analyses to determine the optimal model for the prediction of neonatal mortality and severe neonatal morbidity. In this analysis, mortality and severe morbidity had the birth weight Z score as significant variable (F test 12.33, p-value 0.001 for mortality, and F test 8.26, p-value 0.006 for severe morbidity). Absent or reversal end diastolic flow in umbilical artery, abnormal ductus venous S/A ratio and absent or reversal flow during atrial systole in ductus venous were excluded for the optimal model for both outcomes (Table 1). There was no mortality for the group with normal birth weight Z score.

**Table 1:** Results of backward stepwise logistic regression analysis for neonatal outcome.

***Outcome variable***	***Significant variable***	***F test***	***p-value***	***Excluded variables***
Mortality	-Birth weight Z score	12.33	0.001	-Absent or reversal end diastolic flow in umbilical artery. -Abnormal ductus venosus S/A ratio -Absent or reversal flow during atrial systole in ductus venosus.
Severe Morbidity	-Birth weight Z score	8.26	0.006	-Absent or reversal end diastolic flow in umbilical artery. -Abnormal ductus venosus S/A ratio. -Absent or reversal flow during atrial systole in ductus venosus

The Z score index for the prediction of mortality calculated by the area under the ROC curve was 0.956, standard error 0.029 and p-value < 0.001. The severe morbidity outcome had an index of 0.789 calculated with standard error 0.067 and p-value 0.001 (Table 2).

**Table 2:** Z score birth weight index for the prediction of neonatal outcome.

***Outcome variable***	***AUC***	***SE***	***p-value***
Mortality	0.956	0.029	<0.001
Severe Morbidity	0.789	0.067	0.001

AUC – Area under ROC curve. SE – Standard error.

## Discussion

Although there are many underlying etiologies, IUGR resulting from placental insufficiency is most relevant clinically because outcome could be altered by appropriate diagnosis and timely delivery. It is important to analyze which fetus is at risk and which is the parameter to be considered for timely delivery. Fetal growth restrictions are a physical sign rather than a single disease [13]. Noninvasive antenatal surveillance tools, such as Doppler ultrasound are limited.

Multivessel Doppler surveillance is usually used in the assessment of the fetus at risk. The goal of fetal surveillance in high risk fetuses is to balance fetal and neonatal risk to optimize the timing of intervention. Deterioration of uteroplacental function is initially reflected by abnormal blood flow in the arterial Doppler and ongoing compromise is manifested by abnormal venous Doppler [3,14]. There is a clear association between severe degrees of umbilical Doppler abnormalities, such as absent or reverse end-diastolic velocities and poor pregnancy outcome. Based on previous studies, we assumed that cerebroplacental ratio is potentially more advantageous in predicting outcome [15]. The fetus with abnormal cerebroplacental ratio is usually the one that might benefit from timely appropriate management.

Multivessel Doppler assessment is not absolute and additional information is necessary to estimate the neonatal prognosis. The relationship between abnormal arterial and venous Doppler findings and neonatal outcomes is not well clarified [16,17]. It is important to identify the fetus at risk for adverse neonatal outcome to intervene appropriately and to avoid over treatment and unnecessary fetal and maternal risk.

## Conclusion

Our study has demonstrated that pathological Doppler findings in conjunction with fetal weight Z score index can identify the fetus at risk for neonatal mortality and morbidity. We speculate that Z score index can result in significant clinical improvement to predict the outcome of severe placental insufficiency.
